# Strategic niobium integration and thermomechanical processing in the advancement of novel CMnSiAlPMo TRIP-aided bainitic steel

**DOI:** 10.1038/s41598-026-38448-0

**Published:** 2026-02-24

**Authors:** Hoda Refaiy, Eman El-Shenawy, Jukka Kömi, Mohammed Ali

**Affiliations:** 1 Plastic Deformation Department, Central Metallurgical Research and Development Institute (CMRDI), El-Felzat St, P.O. 11722, Cairo, Egypt; 2 Materials and Mechanical Engineering, Centre for Advanced Steel Research, University of Oulu, P.O. Box 4200, Oulu, FI-90014 Finland; 3 VTT Technical Research Centre of Finland Ltd, Visiokatu 4, P.O. box 1300, Tampere, 33101 Finland; 4 Steel Technology Department, Central Metallurgical Research and Development Institute, Helwan, Cairo, 11421 Egypt

**Keywords:** Austempering, Bainitic steel, Nb microalloying, Retained austenite, Thermomechanical controlled processing, And TRIP-aided steel, Engineering, Materials science

## Abstract

This study examines the effects of niobium (Nb) addition and different thermomechanical controlled processing (TMCP) regimes on the flow stress behaviour and microstructure evolution of a newly developed CMnSiAlPMo TRIP-aided bainitic steel. TMCP tests were conducted with various hot deformation passes, followed by austempering at 400 °C for 10 min using a Gleeble 3800 thermomechanical simulator. Microstructures were analysed using scanning electron microscopy with electron backscattering diffraction and X-ray diffraction. Results showed that increasing the number of passes and reducing the final deformation temperature (FDT) enhanced the flow behaviour for both 0Nb and 0.05Nb alloys, with strain hardening being the dominant mechanism across all regimes. The four-pass regime with an FDT of 850 °C for the 0Nb alloy achieved the highest hardness (457 HV), attributed to grain refinement, which was more influential than the retained austenite fraction. For the 0.05Nb alloy, the two-pass regime at 1050 °C showed the highest hardness (428 HV), resulting from a lower retained austenite fraction. Additionally, Nb addition significantly refined the microstructure and increased the peak flow stress from 385 MPa to 421 MPa for the four-pass regime. The prior austenite grain size decreased from 23 to 12 μm in the single-pass regime, and the largest grain size in the cumulative grain size distribution (D_90%_) decreased from 8.45 to 7.49 μm.

## Introduction

Third-generation advanced high-strength steels (AHSSs), such as TRIP-aided bainitic and quenched-partitioned steels, are essential for reducing greenhouse gas emissions and enhancing automotive fuel efficiency and performance^1,2^. They provide a higher strength-to-weight ratio and superior mechanical properties compared to the first-generation of AHSS, while being more cost-effective than second-generation AHSS^1,2^. Third-generation AHSS supports sustainability goals by reducing reliance on critical alloying elements, enhancing recyclability, and lowering life-cycle emissions^3,4^. Their exceptional strength–ductility balance makes them ideal for automotive structural components such as A- and B-pillars, bumper systems, seats, doors, and other safety-critical parts, where they minimize intrusion into the passenger compartment and effectively absorb impact energy^5,6^.

TRIP-aided bainitic steel (TBS), which contains retained austenite (RA), is known for its high strength and excellent fatigue and impact resistance^7,8^. The metastable RA in TBS improves its mechanical properties through the transformation-induced plasticity (TRIP) effect^9,10^. Optimizing the microstructure and mechanical characteristics of TRIP steels requires both controlling the chemical composition and thermomechanical controlled processing (TMCP) parameters^11–14^. The alloying elements improve phase stability and refine microstructures, resulting in increased strength^14^, while TMCP provides direct control over processing conditions to achieve fine-grained microstructures and balanced mechanical characteristics^15,16^. The development of improved TRIP steels with superior performance characteristics may result from the synergistic application of both strategies^17^.

Recently, medium-carbon TBS has shown an excellent balance of strength, ductility, and toughness^10,18^. E. Chandiran et al.^18^ examined two novel medium-carbon bainitic steels (0.53 C-1.55Si-0.48Mn-0.59Cr-0.05Ni and Fe-0.64 C-1.74Si-2.11Mn-0.2Cr-0.15Ni (wt%)), both exhibiting high tensile strength (> 1.6 GPa) and good elongation (> 12%). Adding Si and Al prevents carbide precipitation during TRIP-steel processing and stabilizes austenite with carbon. TRIP steel may contain 1–3 wt% Si and Al^13,19^. Furthermore, adding approximately 0.07% phosphorus to a steel alloy with low silicon (0.5%) enhances RA formation, outperforming phosphorus-free steel^20^. Phosphorus acts as a solid solution hardener, improving tensile strength, elongation, and the balance of strength and ductility.

The medium Mn steels, which contain Mn between 3% and 12 weight%, are a viable option for producing high strength steels with good formability^21^. These steels offer a balance between strength, malleability, and reduced production costs^21,22^. They boost RA formation with appropriate mechanical stability than the RA in the traditional TRIP steels^21–23^. They have outstanding mechanical characteristics compared to conventional TRIP steels because of the larger fraction of the stabilized RA^22^. As previously indicated, increasing the Mn level from 3.3% to 4.7% in the medium Mn steel resulted in a higher percentage of RA (~ 10–30%), increased carbon solubility, and lowered cementite precipitation temperature^24^. Additionally, the crystallographic texture in TRIP-assisted medium-Mn steel has a great effect in enhancing its mechanical behaviour by influencing TRIP activity, strain distribution, and crack propagation^24,25^. Where intercritical annealing at 750 °C during processing formed a strong RA fiber {111}||ND ferrite texture and high-angle grain boundary, enabling extensive strain-induced martensitic transformation and uniform strain accommodation^26^. The addition of Ni to medium-Mn TRIP steel enhances austenite stability by allowing Ni to partition from ferrite to austenite during annealing^27^. The uniform nanostructure of Ni-bearing steel aids in forming stable, film-like RA between bainitic ferrite, with higher carbon content, leading to improved strain hardening and a larger TRIP effect at high strains^28^.

The use of micro-additions such as Mo and Nb in TRIP steel has synergistic effects to enhance mechanical properties while maintaining cost-effectiveness and resource efficiency^29,30^. Nb addition has a great effect on refining austenite grains during reheating, which improves strength and toughness through precipitation hardening and grain refinement. Mo increases hardenability^31^, austenite stability and fosters the production of austenite-rich martensitic-austenite islands in hot-rolled steel, which enhances the steel’s ability to undergo plastic deformation and its toughness at low temperatures^30^. A previous study referred that interaction between the microalloying elements of Nb and Mo in CMnB steel and thermomechanical processing, including different roughing and finishing deformation passes, followed by a quenching step and tempering treatment, boosted the tensile properties, with a yield strength reached 1107 MPa^32^. Moreover, the combined addition of Nb and Mo improves the strength of steel during tempering, which is related to solute drag and precipitation hardening effect^33^. Mo and Nb together reduce the amount of strain-induced ferrite, leading to a more uniform and refined microstructure that promotes the improvement in strength and toughness^29^. The addition of 0.5 wt% Mo in the composition of a direct-quenched steel also achieved a low transition temperature of -95 °C in the as-quenched condition^33^. Liu et al.^34^ investigated the stability of RA at room temperature and the relationship between microstructure and tensile properties in Nb and Mo microalloyed medium manganese TRIP steel. After quenching and tempering (Q&T), the steel demonstrated impressive tensile properties, with ultimate tensile strength ranging from 878 to 1373 MPa and ductility between 18% and 40%. The significant amount of RA contributed to a discontinuous TRIP effect, resulting in excellent tensile performance.

TMCP of TRIP-aided steel offers energy savings and high productivity, potentially eliminating the need for additional heat treatments. TMCP enhances the mechanical properties of steel by refining its microstructure without the need for extra alloying elements. The key factors influencing TMCP for multiphase steels are the rolling conditions and controlled cooling profiles^7,15,35–37^. While many studies have explored the effect of single-pass TMCP on microstructure and mechanical properties, few have examined the impact of multiphase TMCP on TRIP-aided steel. However, multi-pass TMCP provides greater grain refinement and productivity than single-pass processing^7,15,38^.

T. Hojo et al.^7^ studied the effects of one-step thermomechanical processing on the microstructure and tensile properties of ultrahigh-strength TRIP bainitic steel. Their findings showed that thermomechanical processing resulted in a refined bainitic ferrite matrix with smaller prior austenitic grains, packets, blocks, and laths, along with blocky and film-like residual austenite. The steel achieved an ultimate tensile strength of 1122 MPa. Additionally, the impact of two deformation steps at isothermal temperatures during TMCP of low-carbon TRIP-aided steels on prior austenite characteristics and phase combinations was explored. The study found that increasing prior austenite grain size reduced the martensite/austenite (M/A) phase, leading to higher strength without sacrificing ductility. Furthermore, static recrystallization of austenite produced a higher percentage of the M/A phase than dynamic recrystallization^38^.

Despite extensive research on TRIP-aided bainitic steels, the interplay between thermo-mechanical controlled processing (TMCP) parameters particularly multi-pass deformation sequences, varying final deformation temperatures and Nb microalloying remains poorly understood. Most studies examine alloying additions or TMCP routes in isolation, with limited attention to their combined effects on flow stress evolution, grain refinement, and RA stability in CMnSiAlPMo steels. Addressing this gap, the present work offers an integrated analysis of TMCP regimes and Nb addition to elucidate their synergistic role in controlling microstructure and mechanical performance in a newly developed TRIP-aided bainitic steel.

## Materials and methods

### Alloy design and production

The chemical composition of the studied steels is shown in Table 1. The compositions were designed to develop ultra-high-strength TRIP bainitic steel (TBS) with unique mechanical properties. The steels are melted in an induction furnace and then cast in a Y-shaped casting mould, ensuring efficient and uniform filling, reducing defects, and ensuring high-quality casting. After casting, the Y-shaped blocks undergo machining. The heads of the blocks are cut off, and the remaining sections are cut into smaller parts with dimensions 200 mm × 60 mm × 40 mm. These parts then undergo homogenization heat treatment at 1250 °C for 2 h, promoting uniform alloying elements distribution and reducing segregation, followed by hot forging in the temperature range of 1100 –1000 °C. Various specimens with the dimensions 6 mm x 36 mm and 10 mm x 12 mm were machined from the forged rods for the dilatation testing as well as the physical simulation of TMCP, respectively. The non-recrystallisation temperature (T_NR_) is crucial in the TMCP of micro-alloyed steels. It is the temperature below which the austenite phase no longer recrystallises during deformation, which is essential for refining the microstructure and enhancing strength and toughness. T_NR_ is calculated based on the alloy composition and empirical formula in Eq. (1)^39^. By managing the rolling process with the T_NR_, manufacturers can optimise grain size and improve the mechanical properties of high-strength steel^39^.

1 $$\:{T}_{NR}=887+\:464\mathrm{C}+6445\mathrm{N}\mathrm{b}-644\sqrt{\mathrm{N}\mathrm{b}}+500\mathrm{V}+363\mathrm{A}\mathrm{l}-357\mathrm{S}\mathrm{i}+400\mathrm{M}\mathrm{o}-175\surd\:\mathrm{M}\mathrm{o}\:$$

**Table 1:** Chemical composition of the investigated steels.

Steel	C	Mn	Si	Al	Ni	*P*	Mo	Nb	S	*N*	T_NR_ (^o^C)
0Nb	0.37	3.86	0.64	1.00	1.42	0.05	0.24	0	0.019	0.009	1193
0.05Nb	0.37	3.62	0.73	0.91	1.35	0.05	0.24	0.05	0.020	0.008	1308

### Thermodynamic calculations

**Fig. 1:**
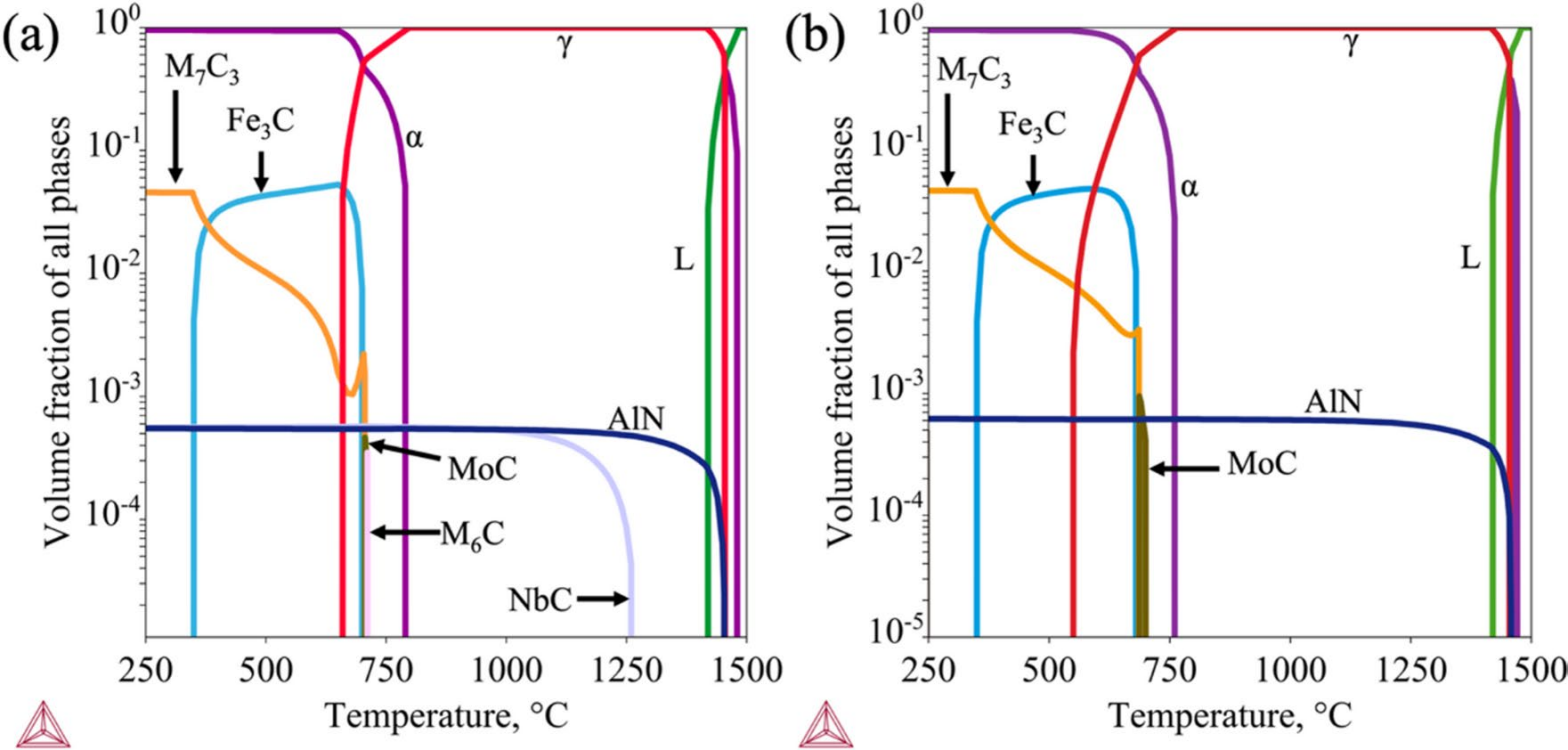
The equilibrium phases predicted by Thermo-Calc software for the designed (**a**) 0.05Nb and (**b**) 0Nb steels.

Thermodynamic calculations were performed using the Thermo-Calc software version 2022a to predict the equilibrium phases and potential carbide/nitride precipitates that could form in the studied steels. Figure 1 shows the predicted volume fraction of phases as a function of temperature for the two steel compositions. AlN forms early at approximately 1450 °C for both alloys, while NbC in the 0.05 Nb steel forms at a temperature of 1250 °C. The behaviour of carbide precipitation, specifically the temperature intervals across which secondary carbides form during cooling, was slightly affected by the addition of Nb. For both alloys, the cementite (Fe_3_C) phase starts to precipitate at about 350 °C and is stable until it completely dissolves at about 700 °C, whereas the M_7_C_3_ carbide is expected to form over a wide temperature range from below 250 °C to near 700 °C.

### Continuous cooling transformation (CCT) diagram

**Fig. 2:**
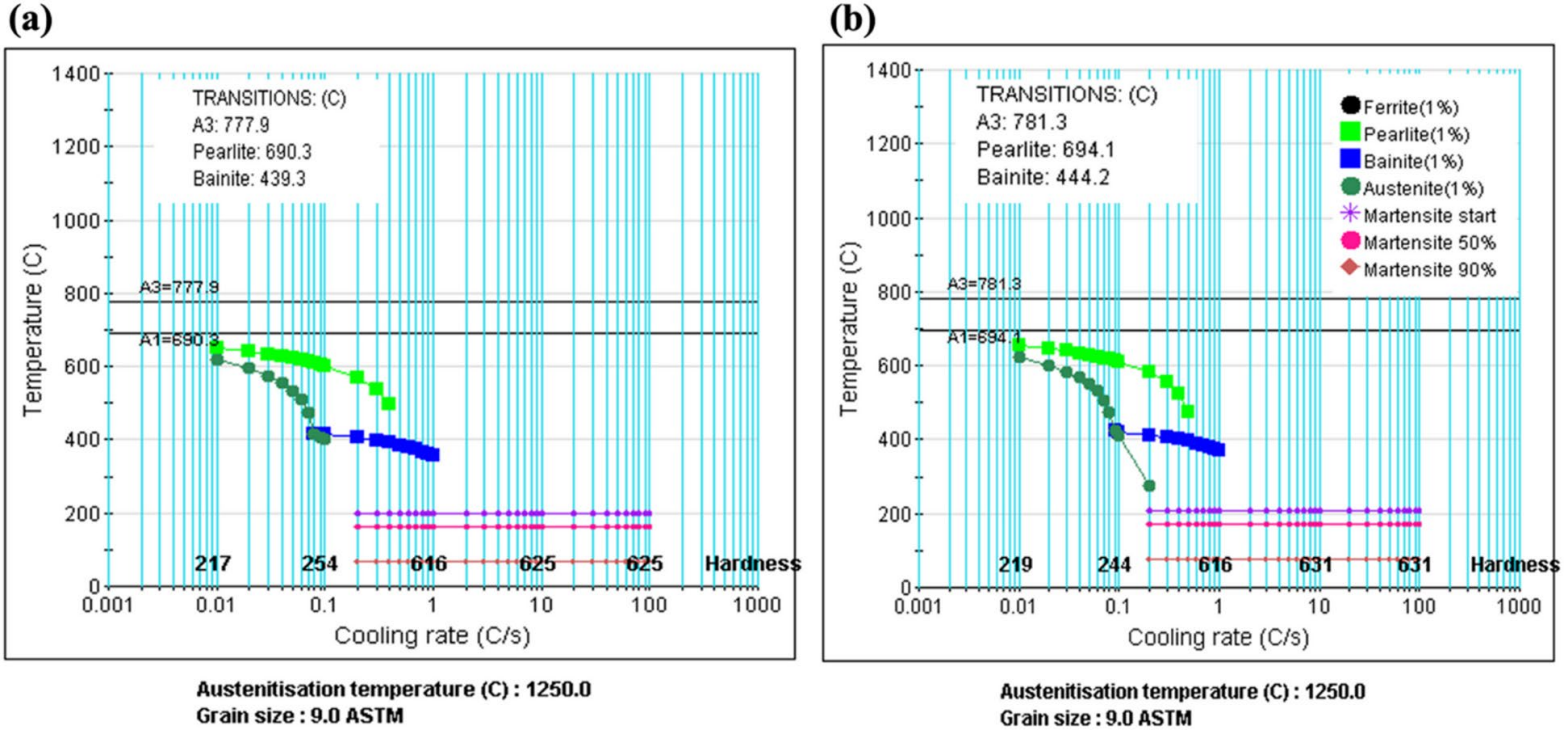
Predicted CCT diagrams for (**a**) 0Nb and (**b**) 0.05Nb steels.

The continuous cooling transformation (CCT) diagrams for the steels under study were calculated using JMatPro software (version 14.0). The CCT curve shows phase transformations during continuous cooling from the austenitic region, helping to optimize heat treatment processes and predict the microstructure and properties of steel under various cooling conditions encountered in manufacturing and service. Figure 2 presents the CCT diagram, highlighting critical cooling rates and temperature ranges related to the formation of microstructural components like pearlite, bainite, and martensite. The curves indicate that the austenite transformation temperature (Ar3) and bainite start temperature (Bs) are around 780 °C and 440 °C, respectively, for both 0Nb and 0.05Nb steels. Notably, martensite is predicted to form at very slow cooling rates (0.2 °C/s).

### Phase transformation and TMCP simulation

#### Phase transformation

**Fig. 3:**
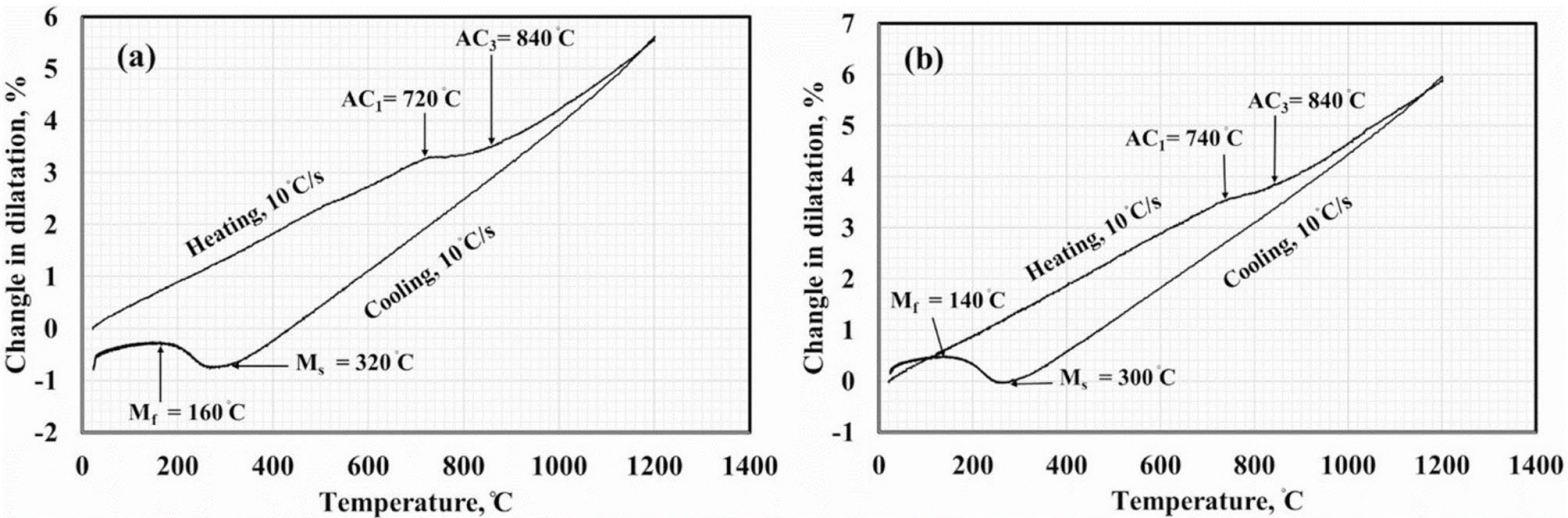
Dilatation curve of steels (**a**) 0Nb and (**b**) 0.05Nb.

A heat treatment cycle was applied to the two steels to determine the actual transformation temperatures. The samples were heated to 1200 °C at a rate of 10 °C/s, held at this temperature for 30s, and then cooled to room temperature at 10 °C/s. Figure 3 displays the dilatation curve of steels where the critical transformation temperatures were identified from abrupt changes in the curve’s slope. During heating, noticeable changes in the curve occurred at 720 °C and 840 °C for 0Nb steel, and at 740 °C and 840 °C for 0.05Nb steel, corresponding to the critical transformation temperatures *A*_*C1*_ and *A*_*C3*_. During cooling, the curve showed significant length reduction, marking the start and finish of the transformation from austenite to martensite (*M*_*s*_ and *M*_*f*_). For 0Nb steel, these are 320 °C and 160 °C, and for 0.05Nb steel, they are 300 °C and 140 °C, respectively. The addition of Nb slightly affected the martensite transformation temperatures, consistent with previous studies that suggest Nb in solid solution lowers the martensite start temperature and promotes ferrite transformation^40^.

#### Gleeble simulation of TMCP

**Fig. 4:**
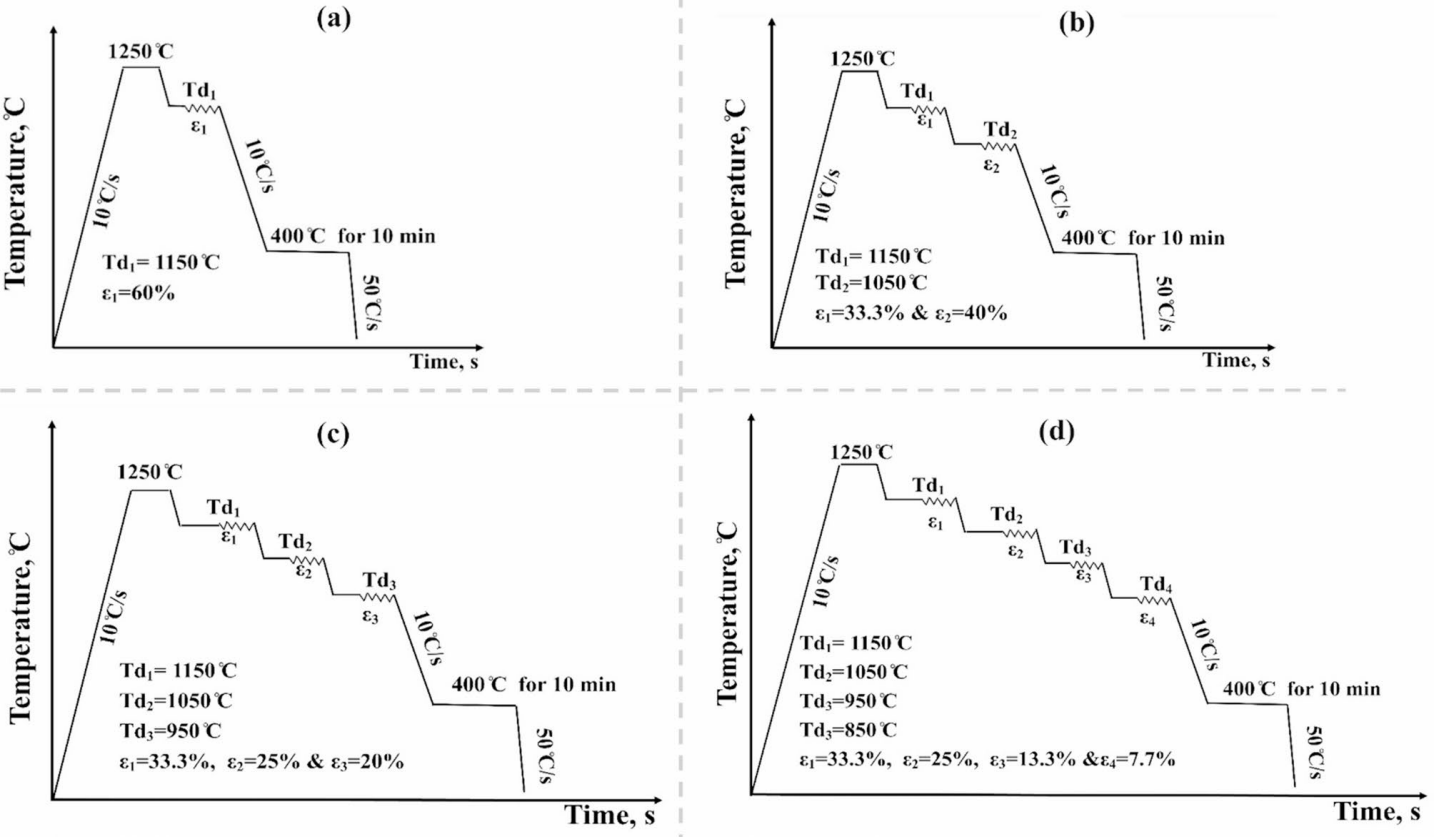
Implemented TMCP cycles (**a**) one pass, (**b**) two passes, (**c**) three passes, and (**d**) four passes, Td: temperature of deformation.

The hot compression test was conducted using a Gleeble 3800 thermomechanical simulator. The initial microstructure of the steels, without deformation or austempering, was examined by heating the samples to 1250 °C for 3 min and cooling them at 10 °C/s. The implemented TMCP cycles for the studied TBS are shown in Fig. 4, with a total reduction of 60% across all regimes. These cycles included austenitization at 1250 °C for 3 min, followed by a one-pass deformation at 1150 °C, denoted by regime 1 (R1), and multi-pass deformation in the 1150 –850 °C range. The cycles involved two passes, three passes, and four passes, denoted by R2, R3, and R4, respectively. After deformation, the samples underwent bainitic transformation at 400 °C for 10 min and were quenched to room temperature at a cooling rate of 50 °C/s.

### Microstructural characterisation

After TMCP, the samples were sectioned and prepared for metallographic examination to evaluate the microstructure. The preparation included mounting the samples in a resin or polymer material, followed by a series of grinding steps using progressively finer abrasive papers and polishing with finer discs. The microstructures were analysed using laser scanning confocal microscopy (LSCM), scanning electron microscopy (SEM) with electron backscatter diffraction (EBSD), and X-ray diffraction (XRD). The samples were etched with 2% nital for LSCM and SEM, while EBSD samples were polished with a 0.04 μm colloidal silica solution.

The volume fraction of RA was determined using X-ray diffraction with a Cu target at 45 kV and 40 mA. RA% was calculated from the average integrated intensity at the (200)γ, (220)γ, (311)γ, (200)α, and (211)α diffraction peaks. Additionally, the carbon concentration in the RA (Cγ) was estimated using the Dyson & Holmes equation (Eq. 2)^13^, and the RA lattice parameter (a_γ_) was measured from the (220) diffraction peak of austenite.

2 $$\:{\mathrm{a}}_{{\upgamma\:}}=3.578+0.033(\mathrm{w}\mathrm{t}.)\:\mathrm{\%}\:{C}_{{\upgamma\:}}$$

The Vickers hardness values of the samples were measured using a Future Tech hardness tester with a 150 kN load.

## Results and discussion

### Effect of Nb on flow behaviour during TMCP

**Fig. 5:**
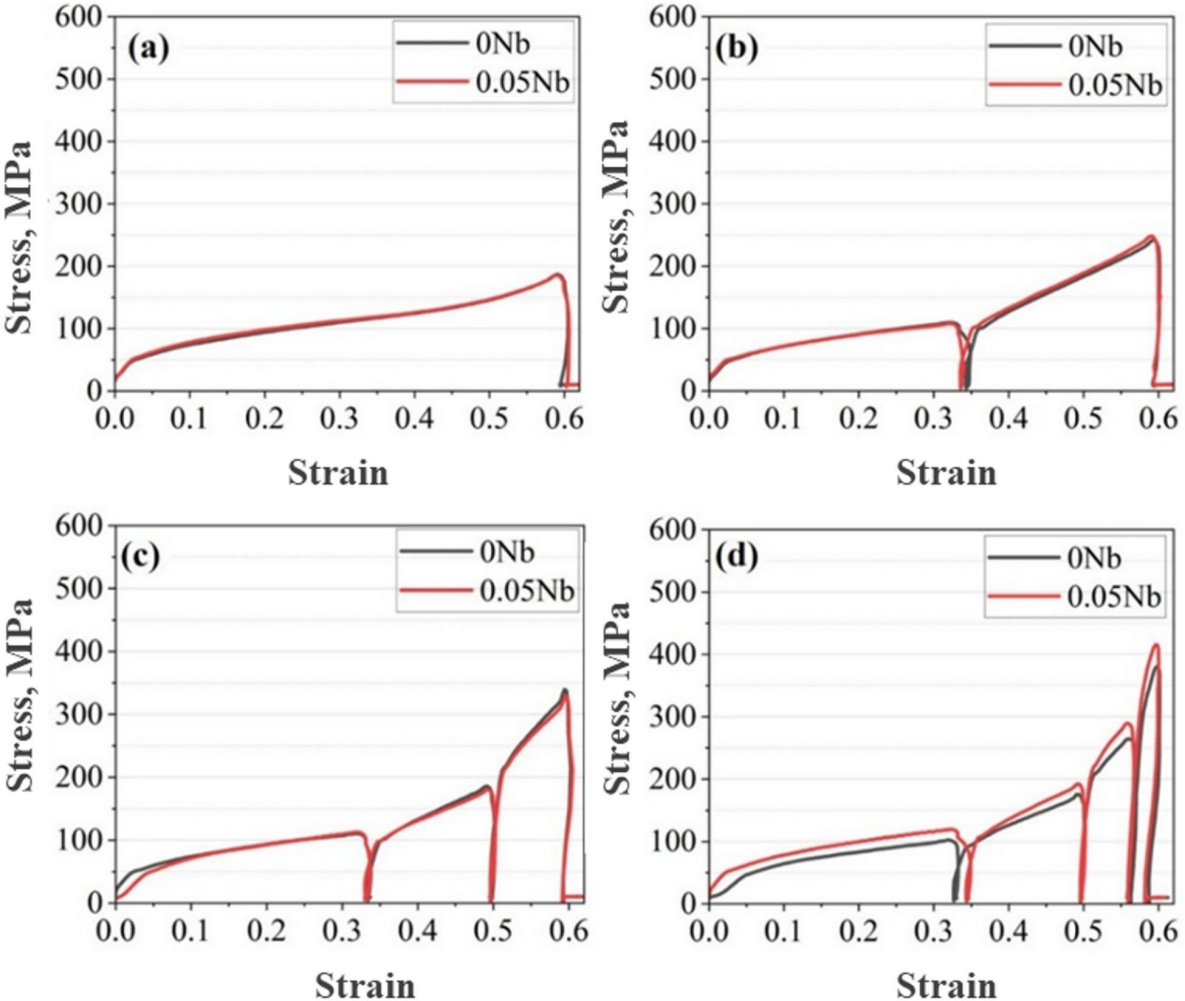
Flow stress-strain curves of the specimens during the physical simulation of different TMCP cycles (**a**) single pass, (**b**) two passes, (**c**) three passes, and (**d**) four passes.

The flow stress-strain curves for 0.05Nb and 0Nb steel specimens during various TMCP techniques are shown in Fig. 5. It is noticeable that the strain hardening is the predominant mechanism for all samples, and increasing the number of deformations passes while keeping the overall percentage of deformation induced a rise in flow stress. Moreover, the Nb addition had no effect on flow behaviour across all regimes. The dislocation density, which is represented in the flow curves, is directly impacted by the simultaneous dislocation creation (strain hardening) and dislocation annihilation (strain softening) that occur during deformation^41^. The build-up of dislocations and their interaction with obstructions results in strain hardening, which raises flow stress quickly^42,43^. Dislocation annihilation and rearrangement cause dynamic softening (DRV and DRX), which causes flow stress to develop more slowly or drop continuously^43^. Furthermore, insufficient static restoration between deformation passes is the cause of the apparent strain hardening in multi-path regimes. Strain hardening accumulates over time because of this partial softening between passes^44^.

### Initial microstructure

**Fig. 6:**
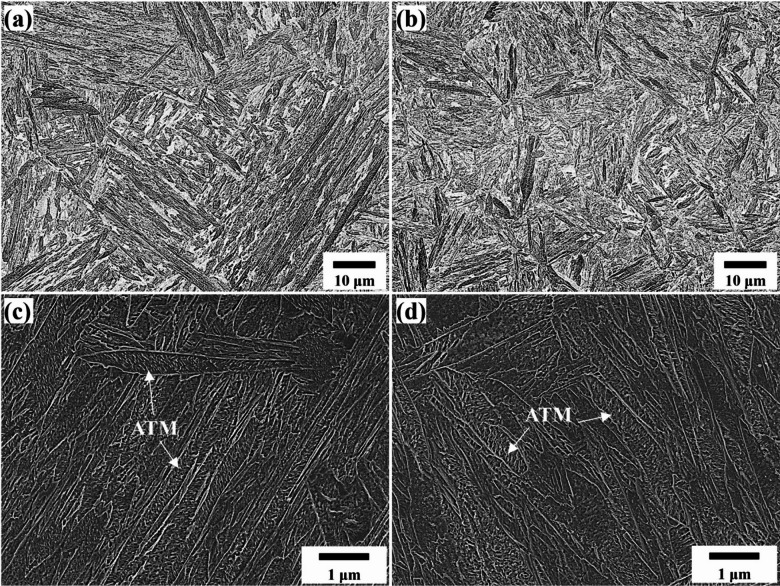
LSCM and SEM micrographs for the initial microstructure of 0Nb (**a** and **c**) and 0.05Nb (**b** and **d**) steels. ATM: Auto-tempered martensite.

Figure 6 shows LSCM and SEM micrographs of the initial microstructure for the 0Nb and 0.05Nb steels. Both steels primarily consist of auto-tempered martensite, consistent with JMatPro results, which predicted that this cooling rate (10 °C/s) after full austenitization promotes martensitic transformation. The addition of Nb further refines the auto-tempered martensite structure. As noted earlier, Nb’s strong affinity for carbon and nitrogen leads to the formation of stable niobium precipitates, i.e., NbC and Nb(C, N), which pin the austenite grains during reheating or austenitization. These resulted in austenite grain refinement and finer martensite substructure upon cooling and phase transformation^45^.

### Microstructure evolution of developed TRIP-aided bainitic steel

This section explores the relationship between applied TMCP and microstructural characteristics, such as PAGS and sub-structure features (EGS and D_90%_). It also covers the restoration mechanisms, supported by microstructural analysis using EBSD.

#### Effect of TMCP and Nb addition on prior austenite grains

The effect of TMCP and Nb addition on prior austenite grain size (PAGS) is critical, as PAGS influences bainite nucleation, growth, and the stability of RA^38,46,47^. Finer austenite grains provide more nucleation sites, promoting a faster and more uniform transformation, which affects the volume fraction and distribution of bainite and RA. Increased grain boundary area also impedes dislocation motion, enhancing work-hardening behaviour^48^. Furthermore, finer grains intensify the TRIP effect during deformation, leading to improved ductility and toughness^38^.

The thermodynamic calculations in Fig. 1 show that the studied steels maintain a fully austenitic phase structure at operating temperatures (900–1250 °C). The fcc-austenitic phase is known to favour dynamic recrystallisation (DRX) during hot deformation^49^, which influences flow stress and microstructural evolution. DRX is a thermally activated, diffusion-controlled mechanism^50^. At high temperatures, processes such as grain boundary migration, dislocation movement, and atomic diffusion are enhanced, promoting DRX in the austenitic structure and affecting the size of both the parent austenite and daughter martensite.

**Fig. 7:**
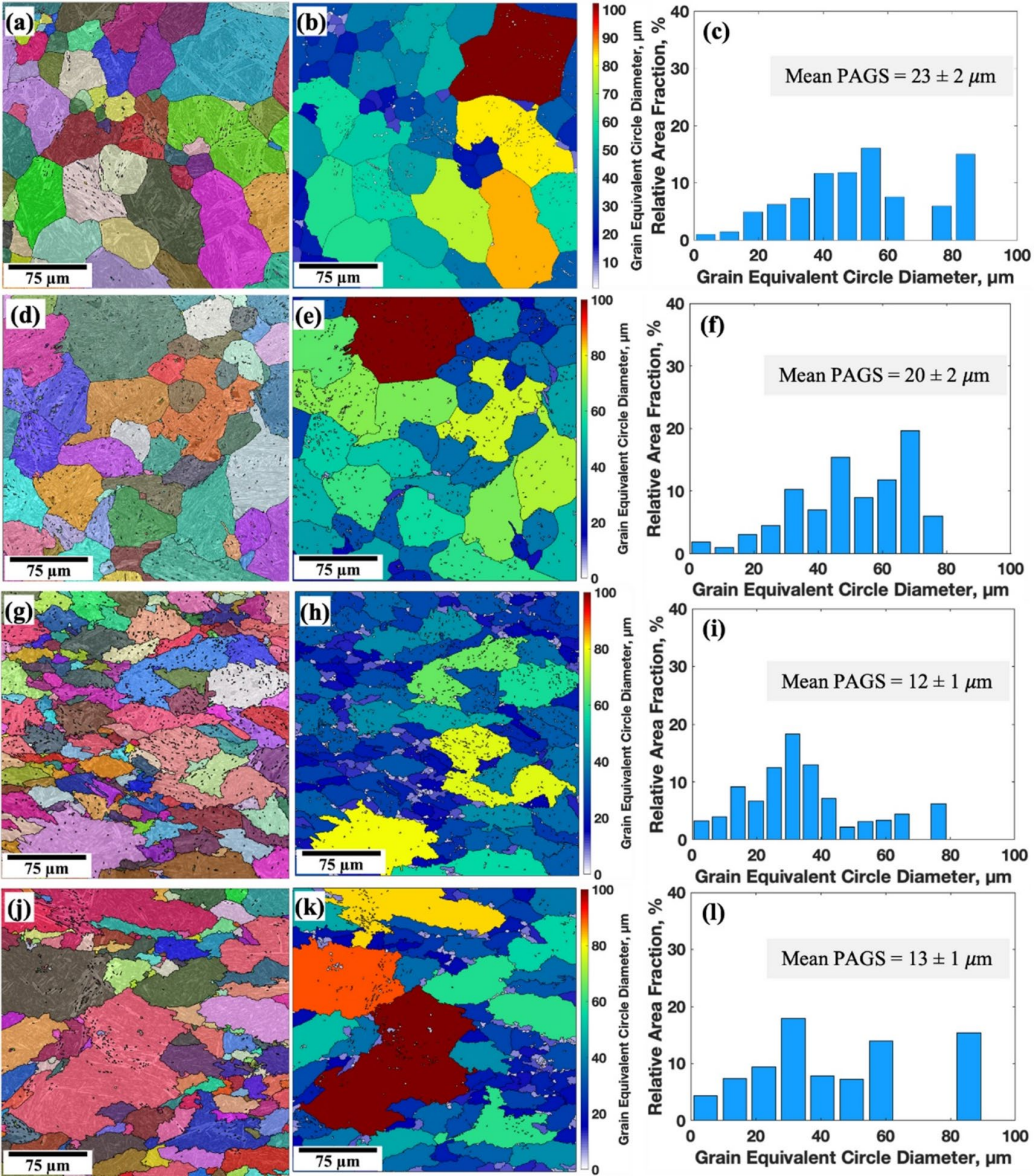
Reconstructed PAGs structures, ECD mapping, and size distribution of PAGs obtained by analysing EBSD data with MTEX and MATLAB software of the simulated samples of 0Nb steel: R1 (**a**, **b**, **c**), R2 (**d**, **e**, **f**), R3 (**g**, **h**, **i**), and R4 (**j**, **k**, **l**).

**Fig. 8:**
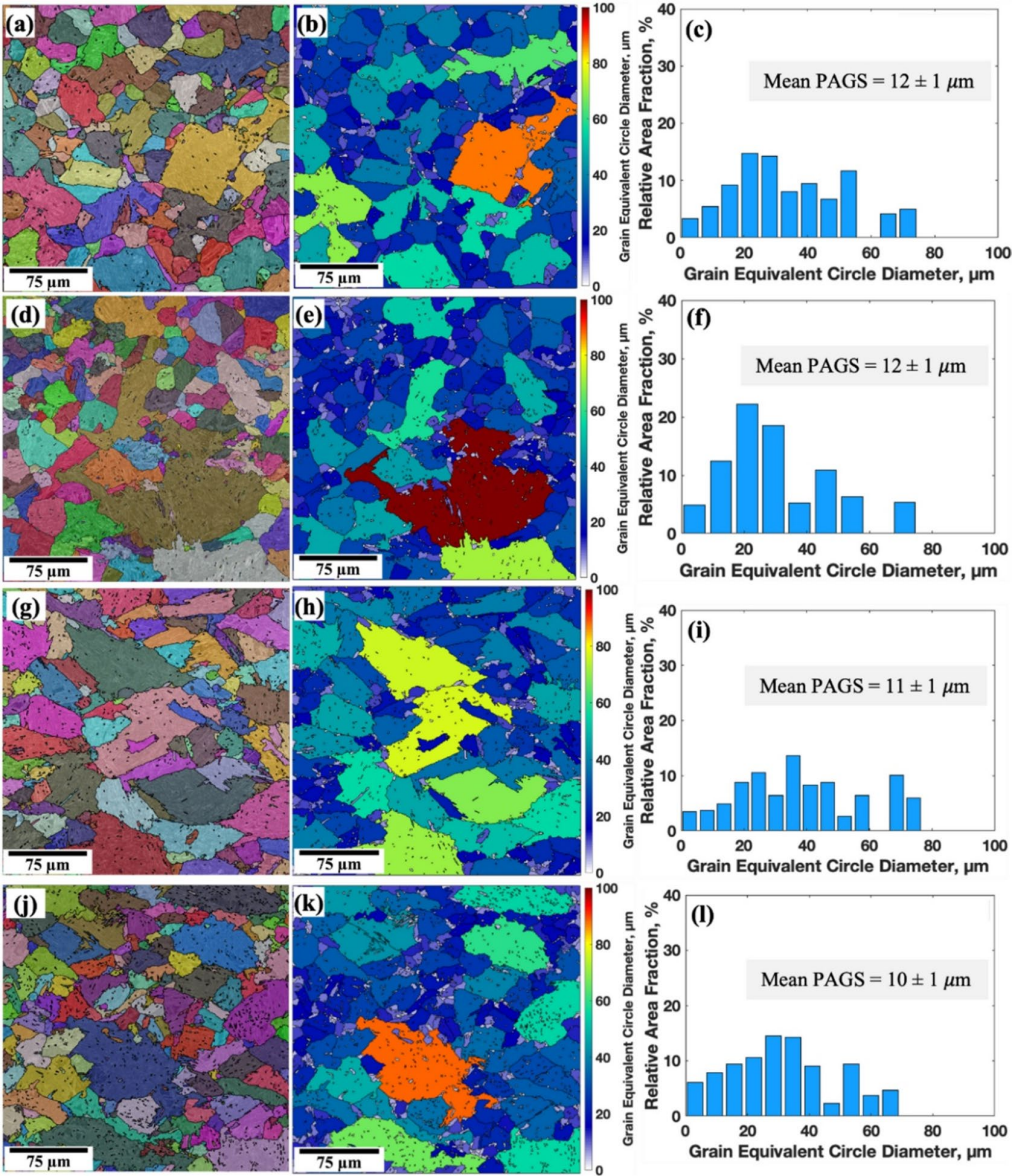
Reconstructed PAGs structures, ECD mapping, and size distribution of PAGs obtained by analysing EBSD data with MTEX and MATLAB software of the simulated samples of 0.05Nb steel: R1 (**a**, **b**, **c**), R2 (**d**, **e**, **f**), R3 (**g**, **h**, **i**), and R4 (**j**, **k**, **l**).

MTEX and MATLAB software were used to reconstruct the prior austenite grains (PAGs) and determine the PAGS of 0Nb and 0.05Nb steels subjected to various TMCP. Figures 7 and 8 exhibit the reconstructed PAGs, the equivalent circular diameter (ECD) mapping of PAGs, and their respective PAGS distributions. Figure 7a displays the PAGs of 0Nb steel after single-pass deformation (R1 regime) at 1150 °C (R1 sample), while Fig. 7b presents the ECD mapping of the PAGs. Complete recrystallisation was observed, followed by grain coarsening due to the high temperature, which increases the thermal activation energy and grain boundary migration. The ECD mapping (Fig. 7b) reveals that most of the grains are below 60 μm in size (in blue and green), although some larger grains exceeding 80 μm (in yellow, orange, and red) are also observed. The size distributions and their mean ECD are shown in Fig. 7c. The mean PAGS is calculated to be 23 μm. Like the R1 sample, complete recrystallisation for R2 regime was observed, followed by grain coarsening. However, two-pass deformation (R2 regime) led to a slight reduction in the size of the PAGs, accompanied by a decrease in the mean PAGS to 20 μm (see Fig. 7d, e,f). Partial recrystallisation was achieved after three-pass deformation (R3 regime), showing mixed very small and large grains. However, compared to the R1 and R2 samples, the R3 sample shows a significant refinement of the PAGs and uniform distribution, and most of the grains´ sizes are below 40 μm, which led to a significant decrease in the mean PAGS to 12 μm (see Fig. 7g, h,i). Four-pass deformation led to a heterogeneous distribution of the PAGs, some of which were non-recrystallised, which appear in very large grains of more than 90 μm, while others are recrystallised and appear in small grains. The average PAGS was calculated to be 13 μm (see Fig. 7j, k,l). This negatively affects the mechanical properties of the produced steels^51^.

Increasing the number of TMCP cycles at low deformation temperatures significantly refines the PAGs, likely due to reduced nucleation rates, grain boundary migration, and slower grain growth at these temperatures^43^. At high temperatures, Nb inhibits grain boundary movement, resulting in a fine-grain structure by preventing recrystallization through the solute drag effect^52^. At low deformation temperatures, NbC or Nb(C, N) precipitates form, further refining the microstructure by pinning grain boundaries^53^. The addition of 0.05% Nb reduced the mean PAGS in all samples (see Fig. 8), with the greatest reduction observed in the R1 and R2 cycles. The mean PAGS decreased from 23 to 20 μm in the 0Nb steel samples (R1 and R2) to 12 μm in the 0.05Nb steel samples (R1 and R2).

#### Effect of TMCP and Nb addition on the general microstructure

**Fig. 9:**
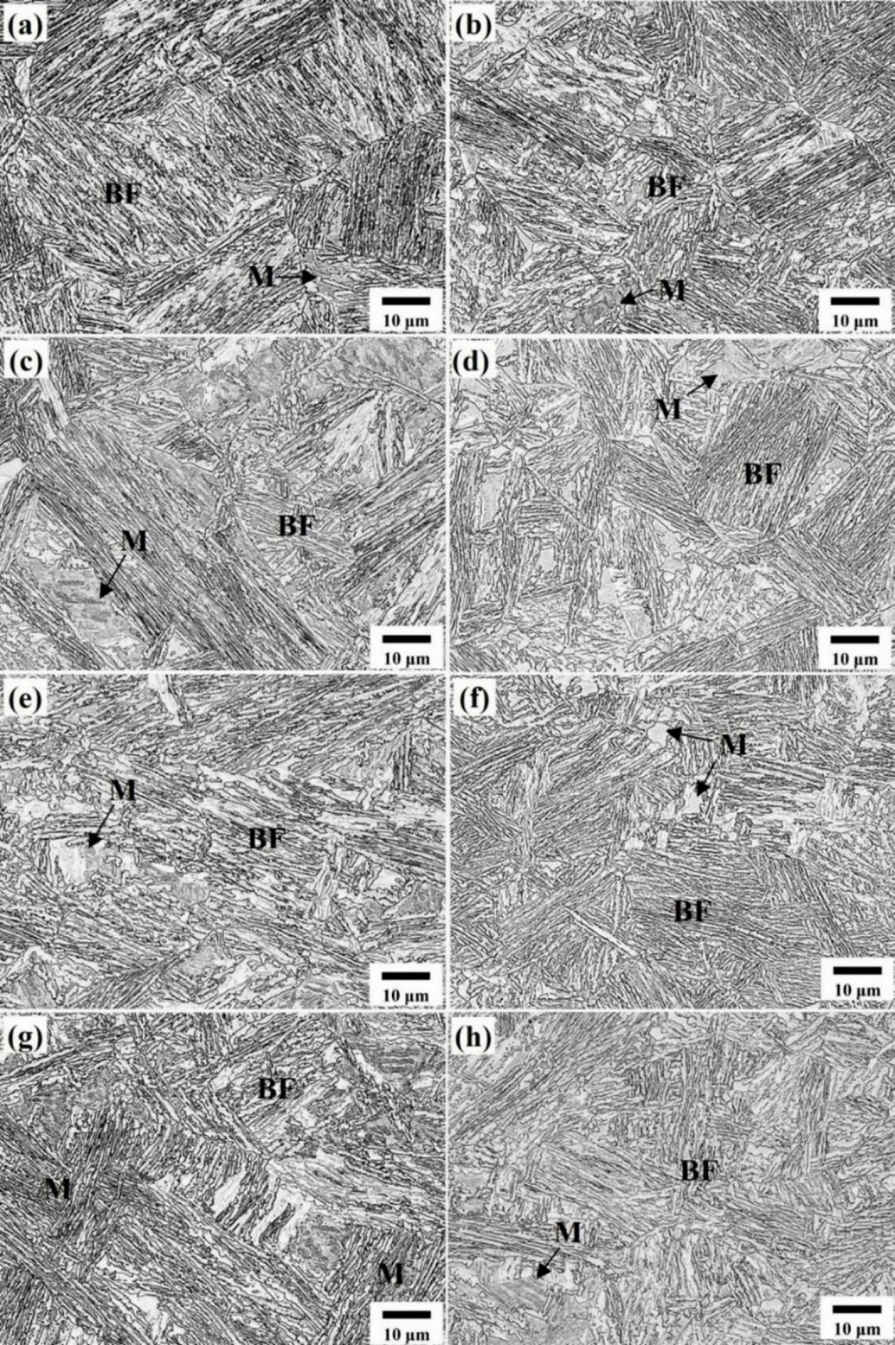
LSCM micrographs of 0Nb (**a**, **c**, **e**, **g**) and 0.05Nb (**b**, **d**, **f**, **h**) after different TMCP regimes R1 (**a**, **b**), R2 (**c**, **d**), R3 (**e**, **f**), and R4 (**g**, **h**). BF: bainitic ferrite, and M: martensite.

**Fig. 10:**
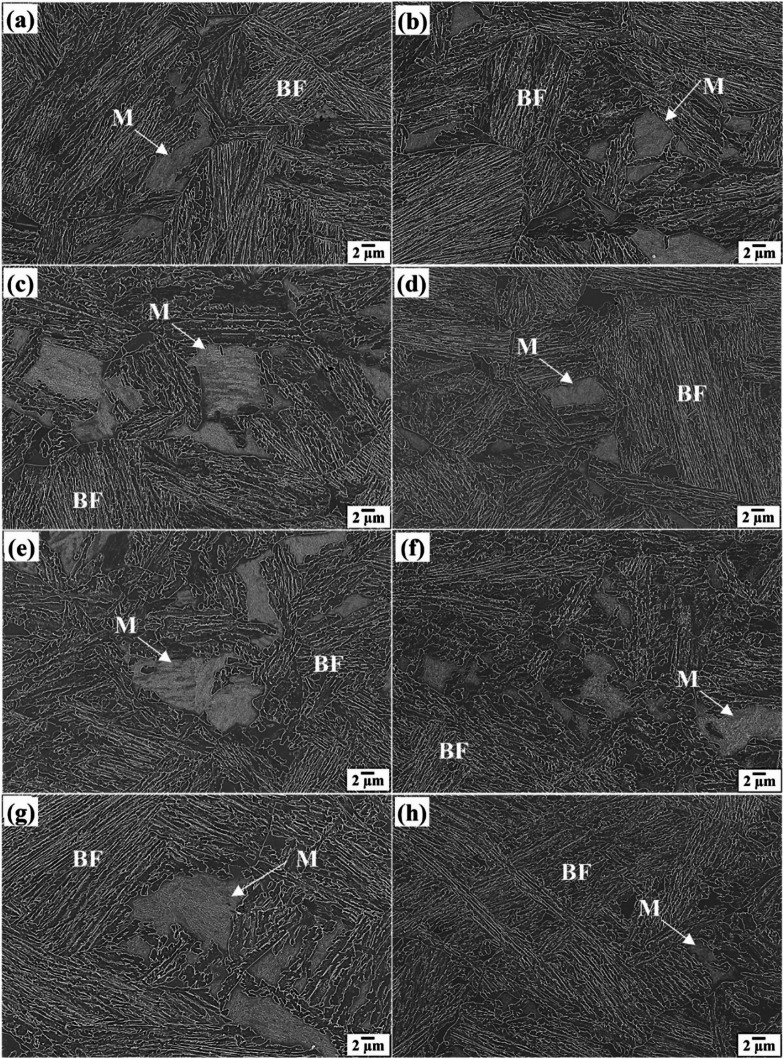
SEM micrographs of 0Nb (**a**,**c**,**e**,**g**) and 0.05Nb (**b**,**d**,**f**,**h**) after different TMCP regimes R1 (**a**,**b**), R2 (**c**,**d**), R3 (**e**,**f**), and R4 (**g**,**h**). BF: Bainitic ferrite, and M: Martensite, RA: Retained austenite.

LSCM and SEM micrographs of 0Nb and 0.05Nb steel samples subjected to different TMCP cycles are shown in Figs. 9 and 10, respectively. The microstructure of both steels after all TMCP consists mainly of bainitic ferrite in addition to blocky martensite and/or austenite, which is particularly noticeable in 0Nb steel. Elevated carbon levels in the remaining austenite after bainitic ferrite transformation can effectively stabilize the austenite phase, inhibiting its transformation into other phases^54^. In the 0.05Nb alloy, the size and quantity of these islands are significantly reduced, especially in the (R4) regime. This suggests that most of the RA exists as inter-lath structures between the bainitic ferrite laths, rather than as blocky islands of austenite. This aligns with previous research, which found that adding 0.04% Nb to a 0.2 C-1.5Mn-1.04Al-0.5Si alloy slowed the transformation of austenite to ferrite, causing most RA to form as layers between the bainitic ferrite laths, while RA in Nb-free alloys exhibited a blocky morphology^55^.

**Fig. 11:**
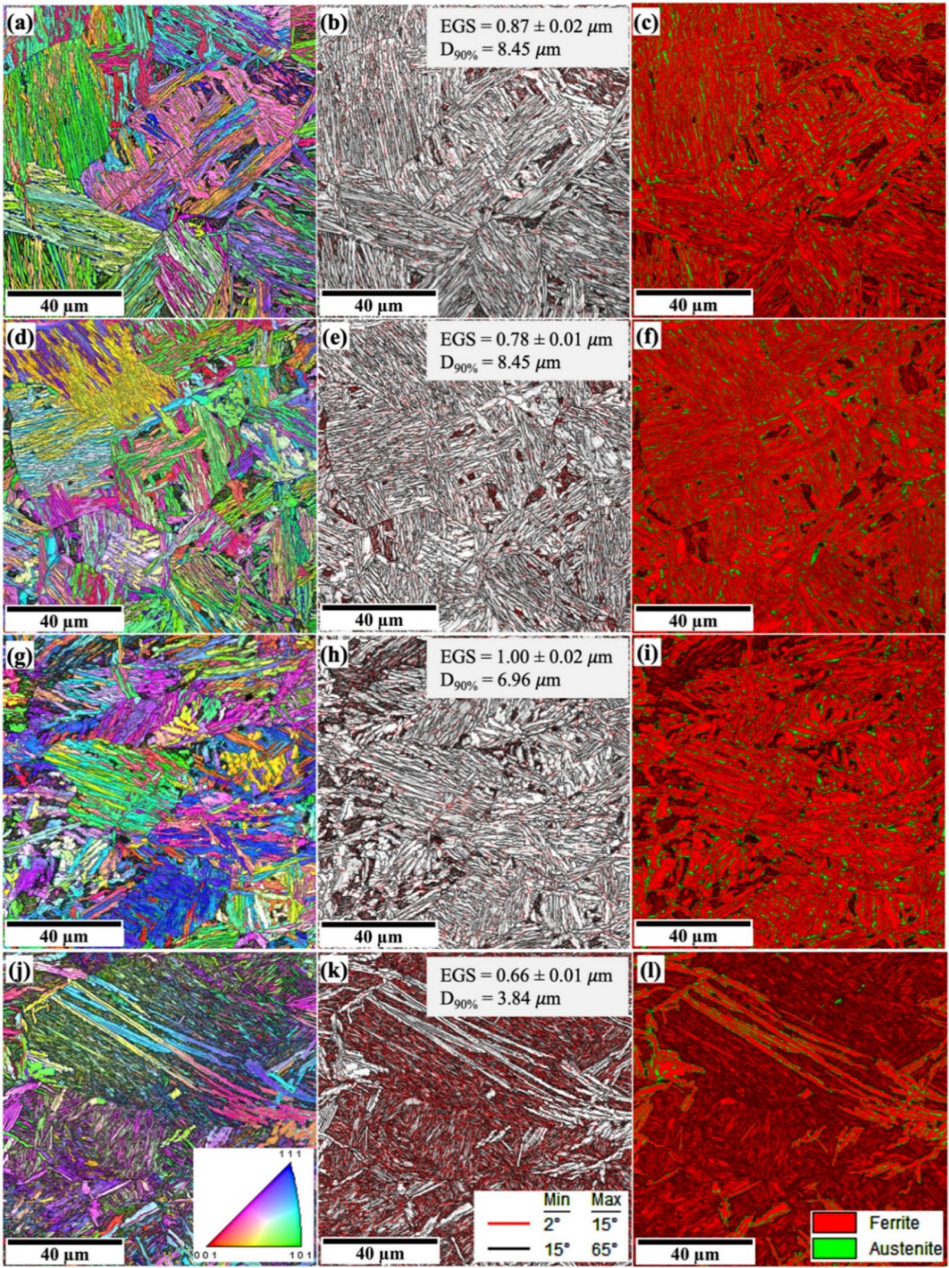
Microstructure of the simulated samples of 0Nb steel: R1 (**a**, **b**, **c**), R2 (**d**, **e**, **f**), R3 (**g**, **h**, **i**), and R4 (**j**, **k**, **l**) recorded at the centreline, (**a**,**d**,**g**,**j**) superimposed inverse pole figure and image quality maps with red = < 001>, green = < 101>, *and blue = < 111>*; (b, e,h, k) grain boundary maps: *low-angle boundaries (red*,* 2–15°)*,* and* high-angle boundaries (black, 15°-65°); (**c**,**f**,**I**,**l**) phase maps.

**Fig. 12:**
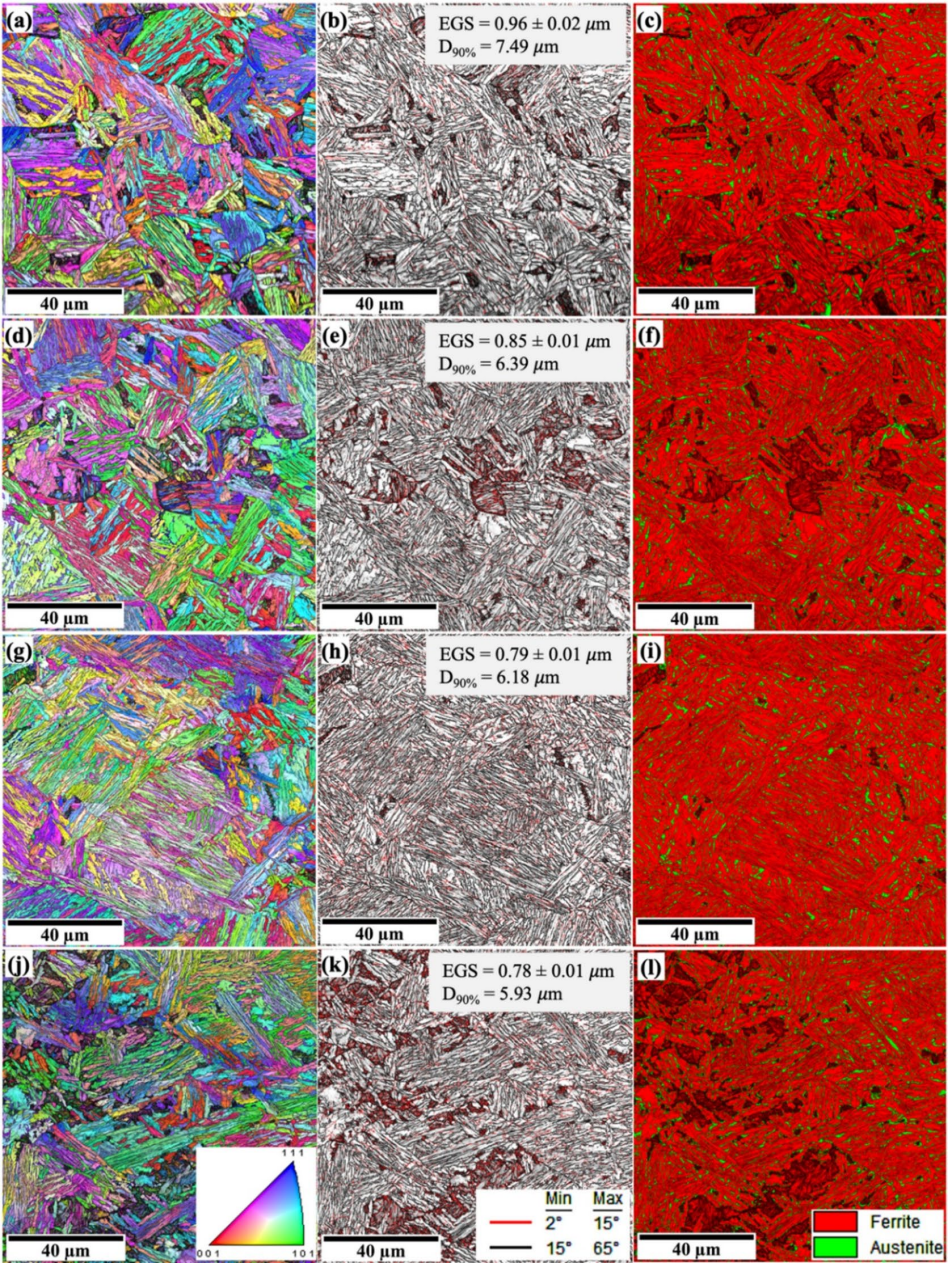
Microstructure of the simulated samples of 0.05Nb steel: R1 (**a**, **b**, **c**), R2 (**d**, **e**, **f**), R3 (**g**, **h**, **i**), and R4 (**j**, **k**, **l**) recorded at the centreline, (**a**,**d**,**g**,**j**) superimposed inverse pole figure and image quality maps with red = < 001>, green = < 101>, and blue = < 111>; (**b**,**e**,**h**,**k**) grain boundary maps: low-angle boundaries (red, 2–15°), and high-angle boundaries (black, 15°-65°); (**c**,**f**,**I**,**l**) phase maps.

The development of secondary phases, such as blocky martensite, is linked to the incomplete partitioning of carbon from bainitic ferrite to untransformed austenite during the austempering process. This observation also aligns with the views on the formation of secondary phases in the microstructure of quenched and partitioned steel, which indicates that the incomplete carbon partitioning from primary martensite to austenite during the partitioning stage has contributed to the formation of these phases^56^. Increasing the number of TMCP cycles at low deformation temperatures significantly refines the microstructure. This is likely due to reduced nucleation rates, grain boundary migration, and slower grain growth at these temperatures^43^. Figures 11 and 12 present the superimposed inverse pole figure, image quality maps, and phase maps for the studied steels. The superimposed inverse pole figure and image quality maps show random orientation of the bainitic ferrite and the martensitic features.

The addition of 0.05 wt% Nb refined the bainitic ferrite and the martensitic features. In R1 and R2 samples, Nb addition has a minor effect on the microstructure features and led to a small refinement of the D_90%_ value (Figs. 11b and e and 12b and e). RA shows uniform distribution in the microstructure of R1 and R2 for both steels (Figs. 11c and f and 12c and f). However, in the case of R3 samples, adding Nb led to a significant refinement of the microstructure features, including EGS and D_90%_. Moreover, the Nb-added steel is nearly fully bainitic ferrite with a uniform distribution of RA, and islands of martensite barely appear in the microstructure. In R4 samples, 0Nb steel provides a fully martensitic structure with a small amount of RA, while 0.05Nb steel shows an inhomogeneous microstructure of bainitic-ferrite/martensite with mixed sizes. This is attributed to the prior austenite grain size, which is still likely to be larger for 0Nb steel than that of the 0.05Nb steel (see Figs. 11l and 12l), even if several passes of deformation somewhat refined the austenite grains. Martensite development during final quenching was encouraged by larger austenite grains, which often result in a higher hardenability. This finding is consistent with other studies showing that finer austenite grains have more grain boundaries, which act as barriers to martensite formation and ultimately lower the Ms temperature^57^.

#### Effect of TMCP and Nb addition on retained austenite

**Fig. 13:**
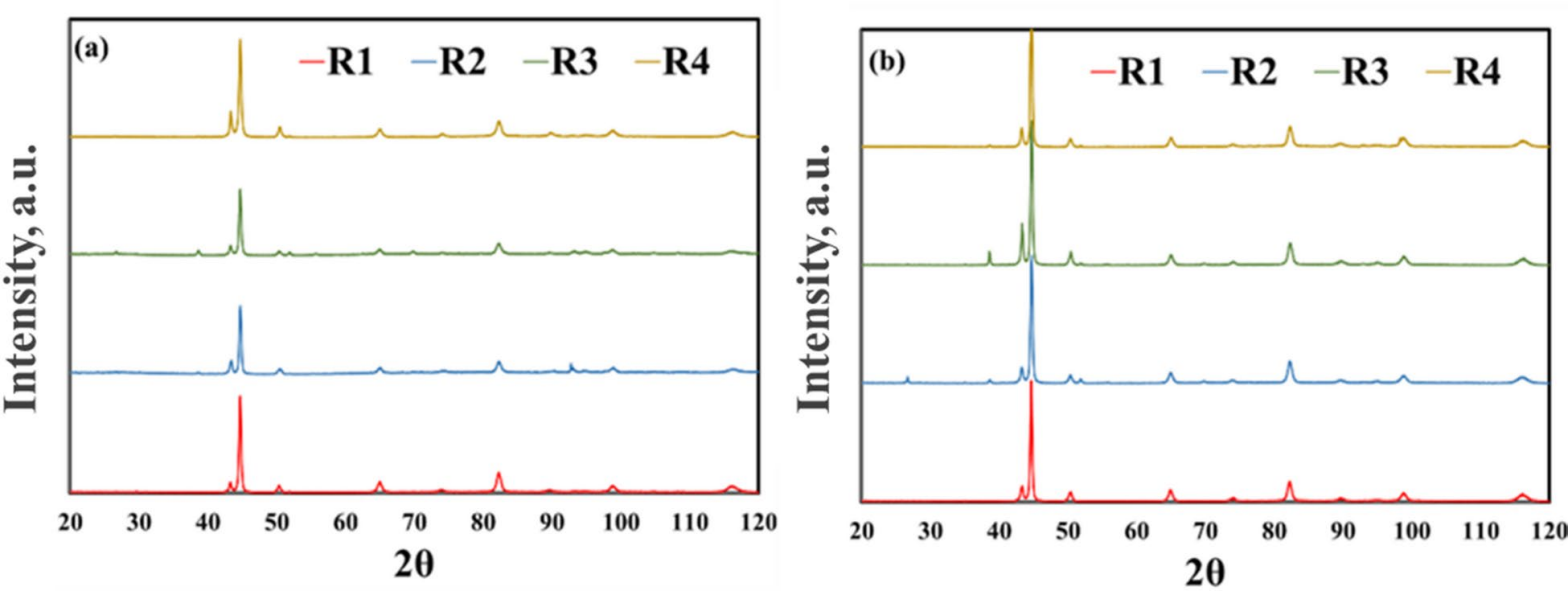
XRD pattern of (**a**) 0Nb steel, and (**b**) 0.05Nb steel samples after different TMCP regimes.

The RA phase plays a vital role in the distinctive mechanical properties of TRIP steels, as it transforms into martensite during deformation, thereby enhancing strain hardening. Figure 13 presents XRD patterns of the studied steels after the application of various TMCP regimes. The volume fraction and carbon content of the RA are determined by analyzing the diffraction peak positions and their integrated intensities for both the face-centered cubic (FCC) structure (austenite) and the body-centered cubic (BCC) structure (ferrite).

**Fig. 14:**
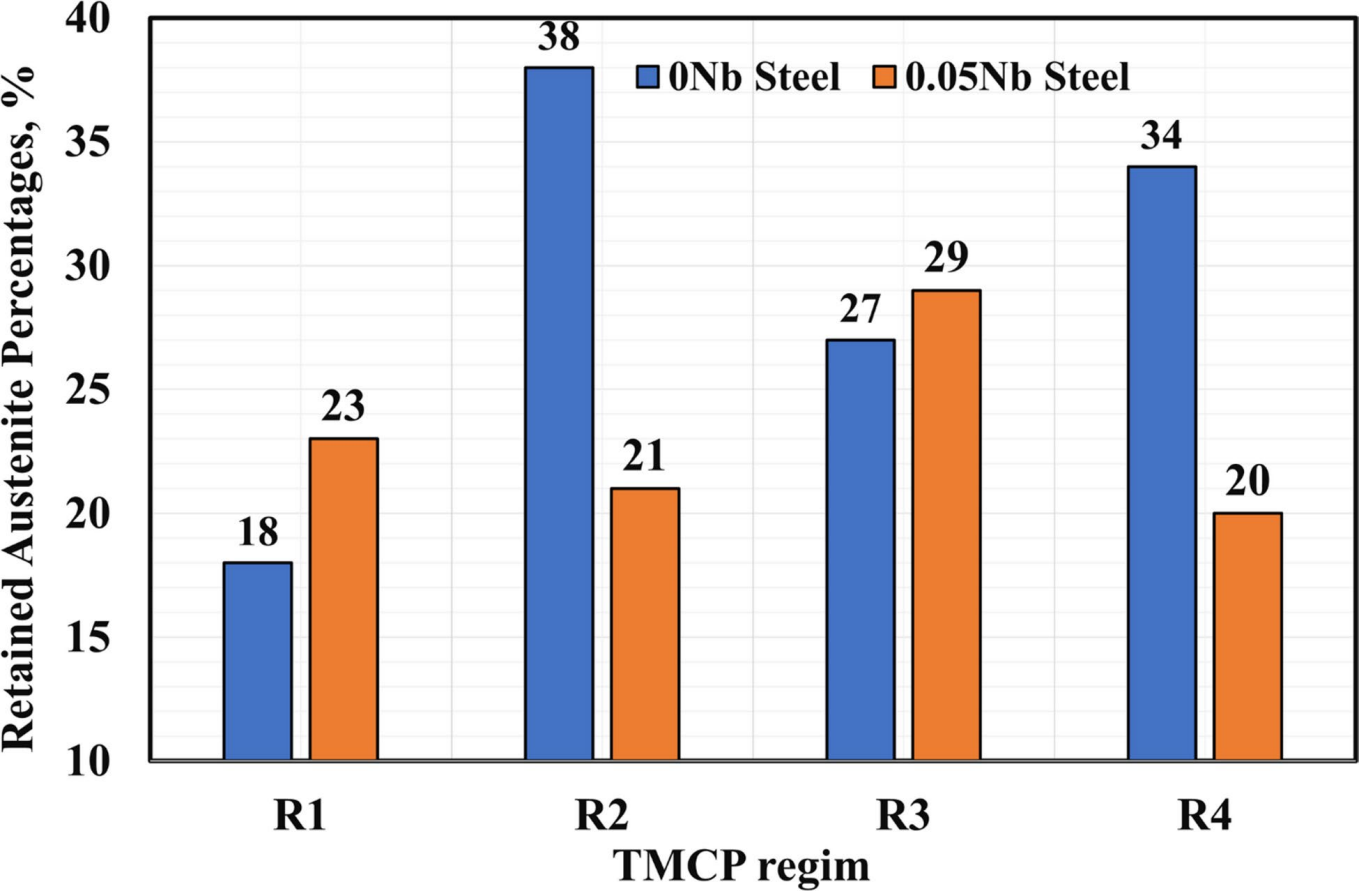
RA percentages in the investigated steels after different TMCP regimes with and without Nb addition.

Figure 14 shows the RA percentages in the investigated steel after different TMCP regimes with and without Nb addition. The 0.05Nb steel demonstrates generally lower RA percentages for R2 and R4 TMCP regimes compared to the 0Nb steel, where the 0Nb steel remains 38% austenite compared to 21% in the 0.05Nb steel. The R1 sample of 0Nb steel achieved the lowest RA fraction of 18% compared to other samples.

Table 2 presents the carbon content of RA in the analyzed specimens. The carbon content of RA in TRIP steel plays a crucial role in determining the phase’s stability and transformation behavior, directly impacting the overall performance of TRIP steels. As presented in Table 2, the carbon content in the RA of the 0Nb and 0.05Nb steels is almost identical for R1 and R3. R2 and R4 samples of the 0Nb alloy exhibit lower carbon content. This decrease is attributed to the formation of a higher volume fraction of RA under these conditions, leading to a lower carbon concentration in each austenite grain.

**Table 2:** Carbon content in the RA of the developed TBS.

Steel code/TMCP regimes	R1	R2	R3	R4
0Nb	1.35	1.14	1.30	1.17
0.05Nb	1.36	1.38	1.28	1.30

### Processing-Properties relationship

**Fig. 15:**
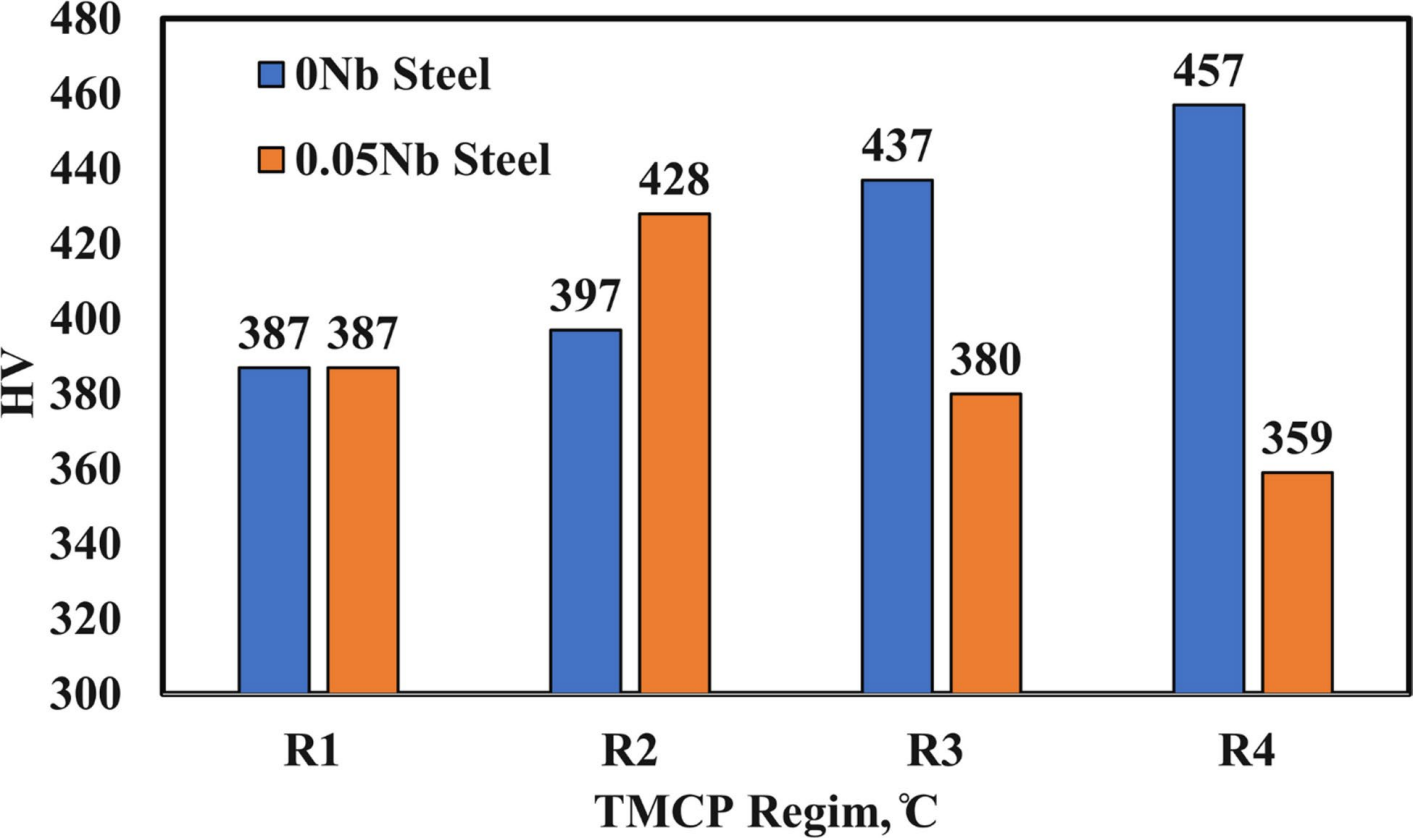
Hardness values of the investigated steels after different TMCP regimes.

The effect of Nb addition and TMCP regimes on the hardness values of the studied TRIP-aided steels is illustrated in Fig. 15. It is evident that the microstructural characteristics produced under each processing condition directly affected the measured hardness values. For the Nb-free steel, hardness gradually increased from 387 to 457 HV as the FDT decreased. This trend is primarily attributed to the grain-refinement effect (see Fig. 11), which played a more dominant role than variations in RA fraction or fluctuations in its carbon content.

For the R1 condition, the hardness values of the 0Nb and 0.05Nb steels were similar despite the refined prior austenite grain size in the latter. This behaviour can be explained by the higher volume fraction of RA stabilized in the Nb-containing steel, which offsets the strengthening expected from grain refinement. Furthermore, similarities in phase size, RA carbon enrichment, and the morphology of additional constituent phases contributed to the comparable hardness outcomes.

In the 0.05Nb steel, the R2 condition yielded the highest hardness (428 HV), which was notably higher than the corresponding R2 value for the 0Nb steel. This response is attributed to the lower RA fraction (21%) in the Nb-bearing alloy compared to the 38% RA stabilized in the Nb-free R2 sample. Conversely, the R3 and R4 conditions of the Nb-containing steel produced lower hardness values than those of the 0Nb alloy. This reduction is associated with the suppression of coarse blocky martensite in the Nb-modified microstructures.

Integrating processing parameters, microstructural features, and targeted mechanical performance provides an effective framework for optimizing TMCP regimes to obtain the necessary attributes in TRIP-aided bainitic steel. This entails achieving a good equilibrium among peak flow stress, RA fraction, carbon content, and hardness. An overview of how different TMCP regimes affect these crucial parameters is shown in Table 3. As previously discussed, a refined matrix together with a higher RA fraction contributes to improved total elongation, strength-ductility synergy, and strain-hardening behaviour in TRIP-aided bainitic steels^10^. There is a strong interrelationship between TMCP parameters, flow-stress response, and the resulting microstructure, particularly prior austenite grain size, phase morphology, RA content, and carbon concentration, all of which collectively govern the hardness.

All TMCP regimes produced multiphase microstructures consisting of bainitic ferrite, blocky martensite, and RA. Among these, the R1 condition exhibited the lowest peak flow stress. This is consistent with the presence of larger prior austenite grains and a reduced RA fraction, which also corresponded with the lowest hardness value (387 HV). For the 0Nb steel, the R2 regime provided a favorable balance of mechanical and microstructural characteristics, including a peak flow stress of 241 MPa, the highest RA fraction (38%), and a moderate RA carbon content (1.14%). Despite these attributes, its hardness remained lower than that of the R3 condition, primarily due to the high RA content, which is comparatively softer.

For the 0.05Nb steel, the R2 regime exhibited a lower RA fraction than R3 but maintained an advantageous microstructure, comprising 21% RA with 1.38% carbon and a reduced peak flow stress of 247 MPa. The R4 regime in the Nb-free alloy achieved the highest hardness (457 HV) along with a 34% RA fraction. However, the large number of deformations passes required at low temperatures significantly increased the peak flow stress and, consequently, the energy and processing time required for TMCP, factors that may limit feasibility in industrial applications.

**Table 3:** Maximum peak flow stress, RA fraction, carbon content, and hardness values of 0Nb and 0.05 Nb steels after different TMCP regimes.

Regime	0Nb Steel				0.05Nb Steel			
	σ_*p*_ (MPa)	RA (%)	Carbon (%)	HV	σ_*p*_ (MPa)	RA (%)	Carbon (%)	HV
R1	168	18	1.351	387	185	23	1.36	387
R2	241	38	1.14	397	247	21	1.38	428
R3	313	27	1.3	437	320	29	1.28	380
R4	385	34	1.17	457	421	20	1.3	359

## Conclusion

This study examines the influence of varying deformation strategies, including a single pass at 1150 °C and multiple passes between 1050 and 850 °C, as well as niobium (Nb) addition, on the flow behaviour, microstructure, and hardness of a newly developed C-Mn-Si-Al-P-Mo TRIP bainitic steel. The key findings are as follows:

1. Strain Hardening and Flow Behaviour:

All thermomechanical controlled processing (TMCP) regimes, with or without Nb, exhibited strain hardening behaviour. An increased number of passes raised the peak flow stress, reaching a maximum of 421 MPa in the four-pass regime of the Nb-containing steel. Moreover, the addition of 0.05% Nb did not affect the peak flow stress compared to the Nb-free steel.

2. Microstructural Characteristics:

All processed samples showed a multiphase microstructure consisting of bainitic ferrite, blocky martensite, and RA. The addition of Nb particularly reduced the blocky martensite content.

3. Retained Austenite Stability:

High RA fractions were observed across all samples, peaking at 38% for the 0Nb steel and 29% for the 0.05Nb alloy. Most TMCP regimes also resulted in high carbon enrichment in RA (~ 1.3%), with minor deviations in the R2 and R3 conditions for the 0Nb steel.

4. Hardness Trends:

For the 0Nb steel, hardness increased with the number of deformations passes (i.e., decreasing final deformation temperature), reaching a maximum of 457 HV in the R4 regime. In the 0.05Nb steel, the highest hardness (428 HV) was recorded in a regime with lower RA content compared to R1 and R3, while hardness declined in R4 despite similar RA levels (~ 20%), likely due to reduced blocky martensite and increased grain refinement.

## Data Availability

The data is provided within the manuscript and can be made available upon request.
